# A population study of clinically actionable genetic variation affecting drug response from the Middle East

**DOI:** 10.1038/s41525-022-00281-5

**Published:** 2022-02-15

**Authors:** Puthen Veettil Jithesh, Mohammed Abuhaliqa, Najeeb Syed, Ikhlak Ahmed, Mohammed El Anbari, Kholoud Bastaki, Shimaa Sherif, Umm-Kulthum Umlai, Zainab Jan, Geethanjali Gandhi, Chidambaram Manickam, Senthil Selvaraj, Chinnu George, Dhinoth Bangarusamy, Rania Abdel-latif, Mashael Al-Shafai, Zohreh Tatari-Calderone, Xavier Estivill, Munir Pirmohamed, The Qatar Genome Program Research Consortium, Rania Abdel-latif, Tariq Abu Saqri, Tariq Abu Zaid, Nahla Afifi, Rashid Al-Ali, Souhaila Al-Khodor, Wadha Al-Muftah, Yasser Al-Sarraj, Omar Albagha, Eiman Alkhayat, Fatima Alkuwari, Hakeem Almabrazi, Mashael Alshafai, Asmaa Althani, Muhammad Alvi, Ramin Badii, Radja Badji, Lotfi Chouchane, Dima Darwish, Ahmed El Khouly, Maryem Ennaifar, Xavier Estivill, Tasnim Fadl, Khalid Fakhro, Eleni Fethnou, Mehshad Hamza, Said I. Ismail, Puthen V. Jithesh, Mohammedhusen Khatib, Wei Liu, Stephan Lorenz, Hamdi Mbarek, Younes Mokrab, Tushar Pathare, Shafeeq Poolat, Fatima Qafoud, Fazulur Rehaman Vempalli, Chadi Saad, Karsten Suhre, Najeeb Syed, Zohreh Tatari, Ramzi Temanni, Sara Tomei, Heba Yasin

**Affiliations:** 1 College of Health & Life Sciences, Hamad Bin Khalifa University, Doha, Qatar; 2 Research Branch, Sidra Medicine, Doha, Qatar; 3 Hamad Medical Corporation, Doha, Qatar; 4 Qatar Genome Program, Qatar Foundation Research Development and Innovation, Doha, Qatar; 5 Department of Biomedical Sciences, College of Health Sciences, Qatar University, Doha, Qatar; 6 Quantitative Genomics Laboratories, Barcelona, Catalonia Spain; 7 Institute of Systems, Molecular and Integrative Biology, University of Liverpool, Liverpool, UK; 8 Qatar Biobank for Medical Research, Qatar Foundation, Doha, Qatar; 9 Institute of Genetics and Molecular Medicine, University of Edinburgh, Edinburgh, UK; 10 Weill Cornell Medicine-Qatar, Doha, Qatar; 11 Weill Cornell Medicine, New York, NY USA

**Keywords:** Pharmacogenomics, Personalized medicine

## Abstract

Clinical implementation of pharmacogenomics will help in personalizing drug prescriptions and alleviate the personal and financial burden due to inefficacy and adverse reactions to drugs. However, such implementation is lagging in many parts of the world, including the Middle East, mainly due to the lack of data on the distribution of actionable pharmacogenomic variation in these ethnicities. We analyzed 6,045 whole genomes from the Qatari population for the distribution of allele frequencies of 2,629 variants in 1,026 genes known to affect 559 drugs or classes of drugs. We also performed a focused analysis of genotypes or diplotypes of 15 genes affecting 46 drugs, which have guidelines for clinical implementation and predicted their phenotypic impact. The allele frequencies of 1,320 variants in 703 genes affecting 299 drugs or class of drugs were significantly different between the Qatari population and other world populations. On average, Qataris carry 3.6 actionable genotypes/diplotypes, affecting 13 drugs with guidelines for clinical implementation, and 99.5% of the individuals had at least one clinically actionable genotype/diplotype. Increased risk of simvastatin-induced myopathy could be predicted in ~32% of Qataris from the diplotypes of *SLCO1B1*, which is higher compared to many other populations, while fewer Qataris may need tacrolimus dosage adjustments for achieving immunosuppression based on the *CYP3A5* diplotypes compared to other world populations. Distinct distribution of actionable pharmacogenomic variation was also observed among the Qatari subpopulations. Our comprehensive study of the distribution of actionable genetic variation affecting drugs in a Middle Eastern population has potential implications for preemptive pharmacogenomic implementation in the region and beyond.

## Introduction

Genetic variation plays an important role in the inter-individual differences in response to medications, and pharmacogenomic (PGx) testing has the potential to provide an informed decision on the appropriate choice and dosage of medications^1^. The current progress in next-generation sequencing (NGS) technologies provides several avenues for PGx profiling. Although many studies have promoted exome sequencing or targeted NGS panels for PGx testing at population scale^2,3^, the benefits of these approaches are mostly limited by their inability to sequence the non-coding regions^4^. Whole-genome sequencing (WGS) can overcome this limitation and hence provide the most comprehensive sequencing approach for more accurate PGx profiling^5^. Furthermore, WGS provides more accurate PGx profiling through the ability to identify potential rare variants/private mutations that may affect drug disposition and response.

Resources such as the PharmVar^6,7^ and Pharmacogenomics KnowledgeBase (PharmGKB)^8^ and guidelines produced by the Clinical Pharmacogenetic Implementation Consortium (CPIC)^9^ and the Dutch Pharmacogenetics Working Group (DPWG)^10^ are helping in the clinical implementation of pharmacogenomic testing for a select number of drug-gene combinations with a high level evidence. However, prioritization and implementation of drug-gene combinations for clinical testing in different ethnic populations require the knowledge of the distribution of genetic variants affecting the drugs and prescription patterns in that population^11–13^. In addition, guidelines developed by CPIC and DPWG primarily focus on common variants, and a WGS approach would help in the identification of novel variants in the population of interest that are currently not covered by CPIC or DPWG. Although pharmacogenomic screening is established in many medical institutions in the US and Europe^14,15^, such implementation is lagging in many other parts of the world, including the Middle East, due to the lack of such data^16^.

Here we present the first comprehensive characterization of clinically actionable genotypes and diplotypes and their predicted phenotypic effect on efficacy, dosing and the risk of adverse events for several medications with CPIC clinical implementation guidelines in the Qatari population from the analysis of 6045 whole genomes. We also compared the distribution of these frequencies with that of other world populations represented in the 1000 genomes dataset to understand the similarities and distinctiveness of the Qatari population in their predicted response to these medications. As far as we are aware, this is the first such comprehensive study in any Middle Eastern population, with potential implications for pre-emptive pharmacogenomic implementation in the region and beyond.

## Results

### Pharmacogenetic variation in the Qatari population

We performed a comprehensive analysis of the variants annotated by PharmGKB to be associated with drugs and based on adjusted p-values from two proportions z-test, the allele frequencies of 1320 variants in 703 genes affecting 299 drugs or class of drugs were significantly different between the Qatari population (6,045 whole genomes) and other world populations represented in the gnomAD v3 dataset (76,156 whole genomes) (Supplementary Data S15). Of these, 615 variants had higher frequencies in the Qatari population. Some examples of variants with differing frequencies in the Qatari population included rs1137101 in the *LEPR* gene, which was lower in the Qataris, rs2289669 in *SLC47A1* and rs11212617 in *ATM*, both higher in the Qatari population. These differing allele frequencies in the Qatari population compared to other populations provides an avenue for further work in the future to determine whether the clinical outcomes are different based on reported drug-gene associations in other ethnic groups^17–19^.

We further performed a focused analysis of the distribution of variants in 17 pharmacogenes affecting 48 drugs, which have CPIC Level A annotation and guidelines for clinical implementation. *UGT1A1* diplotypes were not called with confidence and hence not reported here. Results from *CYP4F2* haplotypes are presented along with warfarin dosage calculations in a later section. Our analysis of the remaining 15 genes affecting 46 drugs identified that, on average, individuals carry 3.6 actionable genotypes/diplotypes. Furthermore, 99.5% of the individuals had at least one clinically actionable genotype/diplotype. Qataris, on average, carried pharmacogenetic variations that predict actionable phenotypes affecting 12.9 (28.8%) drugs.

### Clinically actionable pharmacogenetic variation in the Qatari population

Among the gene-drug pairs with guidelines for clinical implementation, the greatest number of actionable genotypes were present in *VKORC1* (rs9923231; −1639G > A), with 72.7% of the population predicted to require a lower dosage of the widely used anticoagulant, warfarin^20^, based on the homozygous alternate (26.4%) or heterozygous genotypes (46.3%) (Table 1). This was followed by the genotypes of *IFNL3*, which are predicted to produce an unfavorable response to treatment for Hepatitis C in 52.5% of the population studied (Hom alt: 10.4%; Het: 42.2%). Both heterozygous and homozygous alternate genotypes of rs12979860 in *IFNL3* lead to the decreased likelihood of response to pegylated interferon-α and ribavirin therapy^21^. At the other extreme, no variant genotypes were present in the population for *CACNA1S* (rs772226819 and rs1800559), a gene known to cause malignant hyperthermia when using potent volatile anesthetic agents such as halothane or the depolarizing muscle relaxant, succinylcholine^22^. Another related gene, *RYR1* leading to malignant hyperthermia susceptibility^22^ also had only two individuals (0.003%) with the alternate allele for rs111888148 and rs193922762.

**Table 1:** Actionable genotype/diplotype frequencies of clinically important pharmacogenes in the Qatari population.

Gene	Examples of Affected drugs/category of drugs with clinical guidelines	Number of Variants analysed, Number of star alleles analysed	Major Actionable Genotypes/Diplotypes in the population, present in at least 5 individuals in the dataset	Phenotypic effect warranting change in drug, drug dose or drug monitoring	Number of individuals with actionable genotypes/diplotypes in the Qatari population (%)	Total Number of individuals with actionable genotypes/diplotypes in the Qatari population (%)	Total Number of individuals with actionable genotypes/diplotypes in the thousand genome populations (%)
*CACNA1S*	Potent Volatile Anesthetic Agents, Succinylcholine	2	(rs772226819 TT (c.520 C > T), rs1800559 AA (c.3257 G > A))	Malignant Hyperthermia Susceptibility	0 (0)	0	0
*CYP2B6*	Efavirenz	63, 38	*6/*6, *6/*18, *6/*36	Poor metabolizer	497 (8.22)	2781 (46.0)	1295 (51.72)
			*1/*6, *2/*6, *6/*22, *4/*6, *1/*18, *1/*36, *1/*9, *2/*9	Intermediate metabolizer	2284 (37.78)		
*CYP2C9*	Phenytoin, NSAIDs	94, 71	*1/*2, *1/*3, *2/*2, *1/*11, *2/*11, *2/*9,	Intermediate metabolizer	1832 (30.31)	1931 (31.94)	588 (23.48)
			*2/*3, *3/*3	Poor metabolizer	99 (1.64)		
*CYP2C19*	Clopidogrel, Voriconazole, Antidepressants, Proton Pump Inhibitors	71, 39	*1/*17	Rapid metabolizer	1804 (29.84)	3509 (58.05)	1483 (59.23)
			*17/*17	Ultrarapid metabolizer	395 (6.53)		
			*2/*2, *2/*35	Poor metabolizer	113 (1.87)		
			*1/*2, *2/*17, *1/*35, *2/*13, *1/*3, *17/*35	Intermediate metabolizer	1197 (19.8)		
*CYP2D6*	Atomoxetine, Codeine, Ondansetron, Tropisetron, Tamoxifen, Antidepressants	355, 145	*4/*4, *4/*68 + *4, *68 + *4/*68 + *4, *4/*5, *5/*68 + *4	Poor metabolizer	114 (1.89)	2038 (33.71)	982 (39.22)
			*1/*2×2, *2/*2×2, *2×2/*41, *1×2/*2, *1/*1×2, *1×2/*41, *1×2/*1×2, *2×2/*2×2, *1×2/*2×2, *17/*2×2, *2×2/*35, *2×2/*27×2, *2×2/*33, *1×2/*17	Ultrarapid metabolizer	517 (8.55)		
			*1/*4, *1/*68 + *4, *41/*41, *2/*4, *1/*5, *4/*41, *2/*68 + *4, *41/*68 + *4, *10/*41, *2/*5, *17/*41, *41/*5, *17/*4, *4/*10, *35/*4, *1/*3, *10/*68 + *4, *1/*13, *17/*68 + *4, *10/*10, *1/*40, *1/*6, *5/*10, *10/*17, *1/*8, *29/*4, *1/*36 + *10, *1/*7, *1_*2_*68, *17/*17, *17/*29, *41/*9, *9/*68 + *4	Intermediate metabolizer	1407 (23.28)		
*CYP3A5*	Tacrolimus	25, 9	*1/*1	Extensive metabolizer (CYP3A5 expressor)	86 (1.42)	1082 (17.9)	1191 (47.56)
			*1/*3, *1/*6, *1/*7	Intermediate metabolizer (CYP3A5 expressor)	996 (16.48)		
*DPYD*	Fluoropyrimidines			Intermediate metabolizer	9 (0.15)	9 (0.15)	10 (0.4)
HLA-A	Carbamazepine		HLA-A*31:01 Hom	Risk of SJS/TEN	10 (0.16)	333 (5.43)	125 (4.99)
			HLA-A*31:01 Het		323 (5.27)		
HLA-B	Phenytoin, Carbamazepine, Oxcarbazepine		(HLA-B*15:02 Hom)	Risk of SJS/TEN	0 (0)	25 (0.41)	88 (3.51)
			HLA-B*15:02 Het		25 (0.41)		
HLA-B	Abacavir		(HLA-B*57:01 Hom)	Hypersensitivity risk	2 (0.03)	161 (2.62)	151 (6.03)
			HLA-B*57:01 Het		159 (2.59)		
HLA-B	Allopurinol		(HLA-B*58:01 Hom)	Risk of SCAR	4 (0.07)	363 (5.92)	165 (6.59)
			HLA-B*58:01 Het		359 (5.85)		
*IFNL3*	Pegylated Interferon alpha, Ribavirin	1	rs12979860 Hom Alt	Unfavourable response	626 (10.35)	3175 (52.51)	1353 (54.03)
			rs12979860 Het	Unfavourable response	2549 (42.15)		
*NUDT15*	Thiopurines	19, 20	*1/*3	Intermediate metabolizer	245 (4.05)	252 (4.17)	185 (7.39)
			*3/*3	Poor metabolizer	5 (0.08)		
				Indeterminate	2 (0.03)		
*RYR1*	Potent Volatile Anesthetic Agents, Succinylcholine	2	(rs111888148 c.1589 G > A, rs193922762 c.982 C > T)	Malignant Hyperthermia Susceptibility	2 (0.03)	2 (0.03)	0
*SLCO1B1*	Simvastatin	29, 36	*1/*15, *1/*5, *1/*17, *1/*31	Decreased function (Increased risk of myopathy)	1616 (26.73)	1957 (32.37)	376 (15.02)
			*15/*15, *5/*15, *15/*17, *5/*17, *5/*5, *17/*17	Poor function (High risk of myopathy)	341 (5.64)		
*TPMT*	Thiopurines	43, 44	*1/*3, *1/*2	Intermediate metabolizer	120 (1.98)	121 (2.0)	194 (7.75)
				Poor metabolizer	1 (0.02)		
*VKORC1*	Warfarin	1	rs9923231 (−1639G > A) Hom (AA)	Lower dosage requirement	1596 (26.39)	4395 (72.68)	1230 (49.12)
			rs9923231 (−1639G > A) Het (GA)	Lower dosage requirement	2799 (46.29)		

*NSAIDs* Nonsteroidal anti-inflammatory drugs, *SJS/TEN* Stevens–Johnson syndrome, toxic epidermal necrolysis, *SCAR* Severe cutaneous adverse reaction.

Comparison of the frequencies of actionable genotypes/diplotypes in the Qatari population (6045 genomes) with that of the thousand genome populations (2,504 genomes). Examples of drugs predicted to have an effect based on CPIC guidelines are also provided.

Among the highly polymorphic genes with actionable diplotypes, predicted CYP2C19 rapid (29.8%), ultrarapid (6.5%), poor (1.9%) and intermediate (19.8%) metabolizers were present in the Qatari population (58%), which are known to affect the metabolism and necessitate prescribing alternate drugs or a change in dosage of clopidogrel^23^, voriconazole^24^, several antidepressants^25,26^ and proton pump inhibitors^27^. For example, CYP2C19 intermediate and poor metabolizers (21.7%) would require the prescription of an alternate antiplatelet therapy such as prasugrel or ticagrelor instead of clopidogrel, especially when treating patients with acute coronary syndromes (ACS) undergoing percutaneous coronary intervention (PCI)^23^. Similarly, CYP2C19 ultrarapid, rapid and poor metabolizers (38.3%) would need prescribing an alternate antifungal agent instead of voriconazole^24^.

Poor (8.2%) and intermediate (37.8%) metabolizers were predicted from CYP2B6 diplotypes (46%), who may have increased risk of adverse events affecting the central nervous system when treated with the HIV type-1 nonnucleoside reverse transcriptase inhibitor, efavirenz. Both CYP2B6 rapid and ultrarapid metabolizers were also predicted to be present in the population, but there is no evidence of these affecting the plasma concentration of efavirenz, though other drugs may be affected^28^. Increased risk of simvastatin-induced myopathy^29^ could be predicted in 32.4% of Qataris from the diplotypes of *SLCO1B1* (Poor function diplotypes: 5.6%; Decreased function diplotypes: 26.7%). Increased risk of toxicity when treated with the anti-epileptic drug, phenytoin, and several nonsteroidal anti-inflammatory drugs (NSAIDs) metabolized by CYP2C9^30,31^ could be predicted from poor (1.6%) and intermediate (30.3%) metabolizer status in 31.9% of the population.

Actionable diplotypes of one of the most important pharmacogenes, *CYP2D6* were observed in 33.7% of the sampled Qatari population (Poor: 1.9%; Ultrarapid: 8.6%; Intermediate: 23.3%). CYP2D6 metabolizes approximately 25% of drugs, including several antidepressants, codeine, atomoxetine, ondansetron, tropisetron, and tamoxifen^25,26,32–35^. CYP3A5 expresser phenotype was predicted in 18% of the population (Extensive metabolizer: 1.4%; intermediate: 16.5%), which has the CPIC recommendation for an increased starting dose of tacrolimus compared to the standard dose for achieving immunosuppression^36^. Only a small proportion of the population may have an increased risk of thiopurine-related leukopenia, neutropenia or myelosuppression as predicted from the diplotypes of *TPMT* (2%) or *NUDT15* (4.2%)^37^. Similarly, *DPYD* diplotypes predicted only 0.1% of the population may be at increased risk of severe or fatal toxicity when treated with fluoropyrimidines^38^.

A high risk of abacavir hypersensitivity was predicted in 2.6% of the population due to the presence of HLA-B*57:01 homozygous or heterozygous genotypes^39^. HLA-B*58:01 diplotypes (homozygous or heterozygous) were present in 5.9% of the population studied, indicating significantly increased risk of allopurinol-induced severe cutaneous adverse reaction (SCAR), which is manifested by Stevens–Johnson syndrome (SJS), toxic epidermal necrolysis (TEN), or drug reaction with eosinophilia and systemic symptoms (DRESS)^40^. An increased risk of SJS/TEN is also predicted due to the presence of heterozygous or homozygous HLA-B*15:02 in 0.4% of the population if treated with phenytoin/fosphenytoin^30^ or carbamazepine/oxcarbazepine^41^. Furthermore, 5.4% of the Qatari population studied have the HLA-A*31:01 heterozygous or homozygous genotypes, indicating further risk of SJS/TEN and other hypersensitivity phenotypes with carbamazepine treatment^41^.

**Fig. 1:**
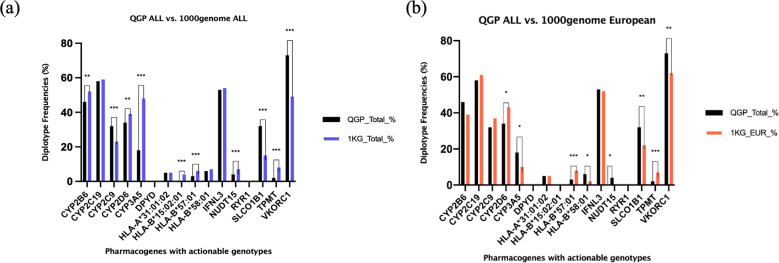
Comparison of actionable genotype or diplotype frequencies in the Qatari population. Actionable frequencies are compared between the Qatari cohort (*n* = 6045) and (**a**) the overall 1000 genomes cohort (*n* = 2504), or (**b**) the European superpopulation in the 1000 genomes cohort (*n* = 503). *p* values of significantly differing frequencies with Bonferroni adjustment indicated as follows: *= 0.0001 ≤ *p* value < 0.05, **= 0.000001 < *p* value < 0.0001, ***= *p* value ≤ 0.000001.

The clinically actionable genotype/diplotype distribution of 10 genes were found to be statistically significantly different in the Qatari population compared to other world populations represented in the 1000 genomes dataset, with only three genes having higher frequencies in the Qatari population (Table 1; Fig. 1a). The actionable diplotype frequency of *SLCO1B1* was more than double in the Qatari population compared to other world populations (QGP: 32% vs. 1000 genomes: 15%, *p* = 3.2 ×10^−59^). Other genes with higher frequencies were *CYP2C9* (32% vs 23%, *p* = 9.2 ×10^−14^) and *VKORC1* (73% vs 49%, *p* = 7.3 ×10^−96^). Furthermore, nine genes had distinctly different actionable frequency distributions between the Qatari population and the European superpopulation from the 1000 genomes dataset, with *CYP2B6*, *CYP3A5*, *NUDT15*, *SLCO1B1*, *VKORC1* and HLA-B*58:01 having higher frequencies in the Qatari population (Table 1; Fig. 1b).

### Clinically actionable pharmacogenetic variation in the Qatari subpopulations

**Fig. 2:**
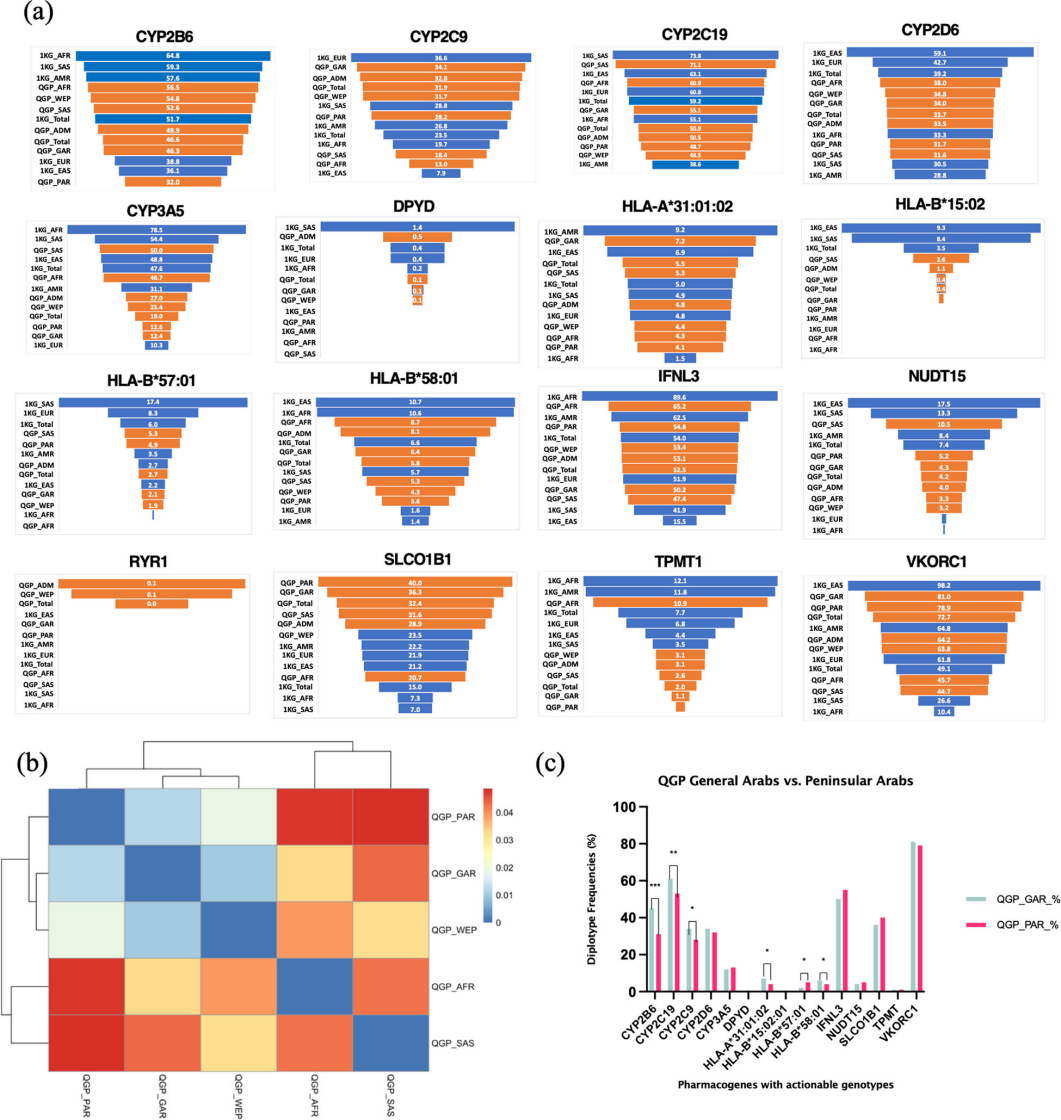
Actionable genotype or diplotype frequencies in the Qatari subpopulations. (**a**) Actionable genotype/diplotype frequencies of clinically important pharmacogenes in the Qatari population and subpopulations (shown as orange bars) along with that of the overall 1000 genomes and the superpopulations (shown as blue bars). (**b**) Clustering of QGP subpopulations based on F_ST_ calculated from the pharmacogenes. (**c**) Comparison of the actionable frequencies of QGP General Arab and Peninsular Arab subgroups. QGP subpopulations PAR, Peninsular Arabs; GAR, General Arabs; ADM, Admixed; WEP, West Eurasian/Persian; AFR: African; SAS, South Asian; 1KG, thousand genomes superpopulations: EUR, European; AMR, American; AFR: African; SAS, South Asian; EAS, East Asian.

Diverse distribution of actionable genotype/diplotype frequencies was observed among the Qatari subpopulations (Fig. 2, Supplementary Data S1–S13). For example, the actionable diplotype frequencies of *CYP3A5* varied from around 12% in the Peninsular Arabs and General Arabs to around 40–50% in South Asian and African subpopulations, with the Persian and Admixed subpopulations having frequencies in the middle of the spectrum (~25%). The Peninsular Arabs had the lowest frequency of actionable diplotypes among the Qatari subpopulations, and the highest in African subpopulation for *TPMT* (PAR: 0.6%, AFR:10.9%), and *CYP2B6* (PAR: 31.3%, AFR: 55.4%). The actionable diplotypes for *DPYD* were totally absent in the Peninsular Arabs, Africans, and the South Asian subpopulations.

Analysis of the fixation index based on the pharmacogenes revealed varying levels of population differentiation between the Qatari subpopulations, with the Peninsular Arabs farther away from the South Asians and Africans, and closer to the West Eurasian/Persians, while being closest to the General Arabs (Fig. 2b). However, the Peninsular Arab subpopulation showed a distinct frequency distribution compared to the General Arabs for some of the genes studied. For example, the Peninsular Arabs had a lower percentage of actionable diplotypes and hence a potentially lower risk of adverse reactions or inefficacy of corresponding drugs when compared with the General Arab subpopulation for *CYP2B6* (31.3% vs. 45.5%,), *CYP2C19* (52.9% vs. 61.2%), *CYP2C9* (28.3% vs. 34.2%), and *CYP2D6* (27.38% vs. 31.76%) while the converse was true for certain other genes: *NUDT15* (5.2% vs. 4.3%), *SLCO1B1* (40% vs. 36.4%) (Fig. 2a, c). Notably, the *SLCO1B1* actionable diplotype frequency for the Peninsular Arabs was the highest among all the subpopulations, which was also almost double that seen in the European, East Asian and American populations (~22%), the highest observed among the populations studied in the 1000 genomes program (Fig. 2a).

Among the HLA alleles, HLA-B*15:02 was completely absent in the Peninsular Arabs, while HLA-A*31:01 was seen at a lower frequency (4.1%) compared to most of the other subpopulations, especially the General Arabs which had the highest frequency in the population (7.2%). The frequency of HLA-B*58:01 was the lowest in the Peninsular Arabs (3.8%) compared to the other subpopulations, with the General Arabs having higher (6.5%) and the African subpopulation having the highest (9%). By contrast, Peninsular Arabs had one of the highest frequencies for HLA-B*57:01 (4.9%) compared to the other subpopulations, including General Arabs (2.1%), and this genotype was absent in the African subpopulation (Fig. 2).

### Warfarin dosing and the potential pre-emptive pharmacogenomic implementation

**Fig. 3:**
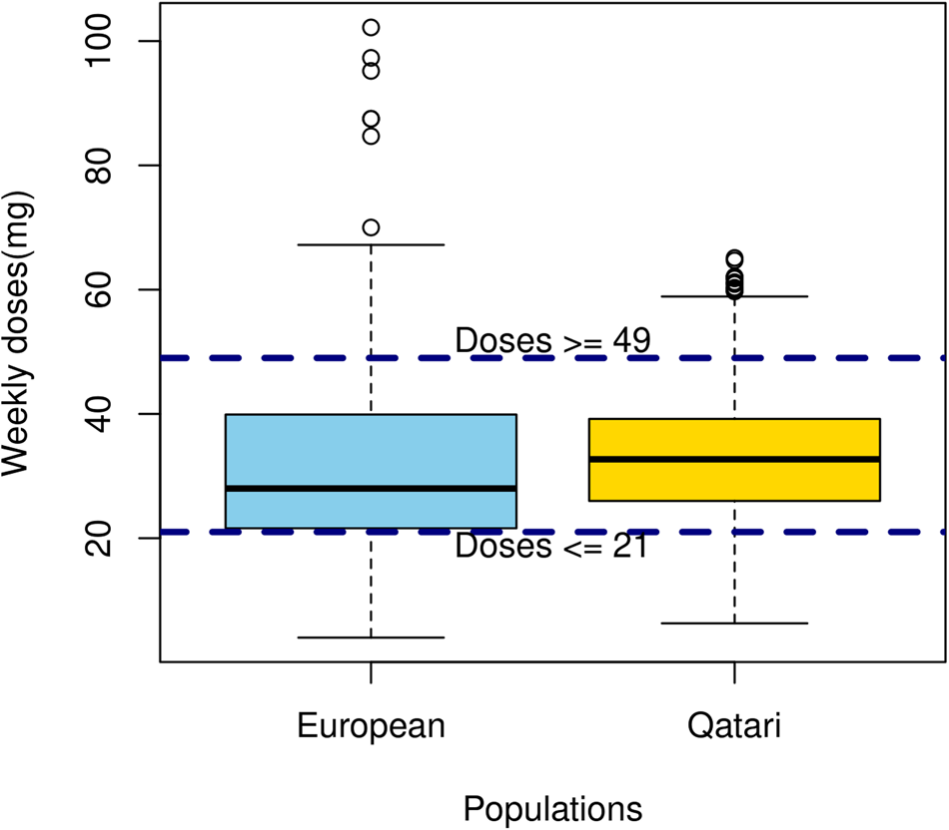
Warfarin dosing prediction. Distribution of the predicted weekly dose (mg) of warfarin in the Qatari population (yellow) using the IWPC algorithm based on age, sex, height, weight, ethnicity, the concurrent use of drugs that decrease warfarin requirements, and the genotypes/diplotypes of *VKORC1* and *CYP2C9*. Also shown is the distribution of dosage in patients of European ethnicity from the EU-PACT trial (blue). Weekly doses in mg are plotted in the Y-axis for the two populations. The box is drawn with the interquartile range and the central horizontal line showing the median, while values above the range shown as whiskers. Individuals with values ≤21 mg per week (below the bottom horizontal line) would be predicted to need a lower dose, and those with values ≥49 mg per week (above the top horizontal line) would be predicted to need a higher dose.

A major concern in the use of warfarin, one of the most commonly used anticoagulants worldwide, is its narrow therapeutic index, which necessitates accurate dosage calculation and therapeutic drug monitoring. We assessed the potential dosage requirements of individuals in the cohort by a combinatorial calculation of the effects of clinical and genetic factors following the IWPC algorithm^42^ that included age, sex, height, weight, ethnicity, and the concurrent use of drugs that alter warfarin requirements, in addition to the genotypes of *VKORC1* and diplotypes of *CYP2C9* as described in the Methods section. The distribution of the predicted weekly dose of warfarin in the population ranged from 5.4 mg to 66.4 mg, with 593 (10%) requiring a lower dose (≤21 mg per week) and 313 (5%) requiring a higher dose (≥49 mg per week). In comparison, in the patients of European ethnicity from the EU-PACT trial (*n* = 325 patients with stable doses), 80 (25%) needed a lower dose and 42 (13%) needed a higher dose (Fig. 3).

In addition to the alleles used for the calculation of warfarin dosage, other *CYP2C9* haplotypes were also present in the population. For example, CYP2C9*11 known to affect dosage in the African population^43^ was seen in the Qatari population (0.5%), with the African subpopulation having the highest frequency (2.7%), close to that seen in the African population in the 1000 genomes dataset (2.3%). CYP4F2*3 haplotype associated with warfarin dosing was present in ~24% of the population, with the lowest frequency in the African subpopulation (8.69%), and the highest in Peninsular Arabs (38.2%). The overall frequency of the CYP2C rs12777823 variant, which is known to lead to a lower dose requirement of warfarin, was ~12%, with the lowest frequency in the Peninsular Arabs (7.8%) and higher frequencies in the African (21.7%) and South Asian (34.2%) subpopulations.

### Medications with actionable genetic variation in the Qatari population

Further to deciphering the landscape of pharmacogenomic variation affecting the CPIC Level A drugs, we also examined the availability of these drugs through the major healthcare provider in Qatar. Out of the 50 drugs, 13 (26%), including abacavir, atazanavir, and ivacaftor were unavailable, while efavirenz was available as non-formulary (Supplementary Data S16).

## Discussion

In this study, we assessed the landscape of clinically actionable pharmacogenomic variation associated with drug response in the Qatari population. One important point to highlight about our study is that, unlike previous studies in the population^44^, we conducted a fine-grained analysis that led to results with a high resolution. For example, rather than just using single variants as proxy to define the star alleles, we used all the variants known to contribute to the haplotype, wherever possible. Furthermore, we analyzed whole-genome sequencing data from ~2% of the Qatari population, capturing broader variant and population landscape than exome studies in small cohorts. Thus, the genotypes and diplotypes we report in the Qatari population can be compared with results from future studies of other populations with confidence. Another aspect of our study is the use of multiple types of variants, for example, structural variants to define the star alleles of *CYP2D6*, unlike some of the recent large-scale studies^45^. This has helped us to capture and present a more accurate account of the distribution of the pharmacogenomic variation in the population. More importantly, we provide a more useful and clinically translatable measure of genetic variability in terms of combined genotype or diplotype frequencies that are actionable based on available guidelines, rather than just reporting allele or haplotype frequencies as done in most studies.

Based on the results from the current study and considering the disease epidemiology and prescription pattern, it is possible to recommend which drug-gene combinations need to be prioritized for pharmacogenomic testing in the hospitals in Qatar and potentially other Middle East countries. For example, the frequency of the actionable *SLCO1B1* diplotypes is high in the population, especially in the Arab subpopulations, suggesting higher risk of muscle toxicity when treated with commonly prescribed lipid lowering HMG CoA reductase inhibitors (statins) such as simvastatin and potentially atorvastatin. Dyslipidemia is the topmost noncommunicable disease (~30%) identified in the QBB cohort and statins are among the highly prescribed medications in Qatar^46^. Thus, for implementation, genotyping of *SLCO1B1* before statins are prescribed or undertaking genotyping in individuals intolerant of statins, should be considered, especially since there are other statins, which have a lower substrate affinity for OATP1B1 could be considered as alternate hypolipidemic drugs^29^.

By contrast, the frequencies of actionable diplotypes of certain other genes were lower in the Qatari population compared to other world populations. A striking example is *CYP3A5* (QGP: 18% vs 1000 genomes: 48%), the lower frequency of which suggests that tacrolimus dosage adjustments may be necessary only in fewer patients for achieving immunosuppression^36^. Of the two genes which can lead to adverse effects when treated with thiopurines, including azathioprine, mercaptopurine and thioguanine, *TPMT* had a lower frequency of actionable diplotypes in the Qatari population (2% vs. 8%), while *NUDT15* had a higher frequency when compared to other populations represented in the thousand genomes dataset (4% vs. 0.2%). *NUDT15* loss-of-function alleles are known to be more prevalent in Asians and Hispanics leading to myelosuppression, rather than TPMT deficiency, which is the primary genetic cause of thiopurine intolerance in Europeans and Africans^37^. Our results suggest that *NUDT15* testing may be more important in the Qatari population than *TPMT* before initiating thiopurine treatment, but in order to improve the safety of thiopurines, we would suggest genotyping for allelic variation in both genes, in keeping with the CPIC guidance^37^.

Genetic variation in the non-coding region also contributes significantly in determining the efficacy or dosage requirements of certain treatments. For example, a non-coding variant in the promoter region of *VKORC1*, c.−1639G > A (rs9923231) alters the transcription factor binding site, leading to decreased expression of vitamin K epoxide reductase enzyme, a target of warfarin. Patients carrying at least one A allele at the −1639 locus require lower initial and maintenance doses of warfarin compared to the patients carrying a G/G genotype^47^. Our study indicates a high frequency of the alternate genotypes in the Qatari population. This example stresses the importance of WGS to identify genetic variation in the non-coding regions, which will be missed by exome sequencing.

Furthermore, *VKORC1* c.−1639G > A and CYP2C9*2 and *3 were found to be the major genetic variants to predict warfarin dosage in a Qatari patient cohort, explaining 39.2% of warfarin dose variability, along with hypertension, smoking and heart failure, while CYP4F2*3 was not associated with warfarin dose^48^. Studies in other populations in the region also showed the significant contribution of these genetic variants in explaining 30–63% of warfarin dose variability, highlighting the clinical importance of PGx-guided warfarin dose recommendations^49–51^. Although many of those populations share the same ancestry and are similar in their warfarin dose predictors, a population-specific dosing algorithm may be better suited for the prospective estimation of warfarin dose. A multivariate model, which included demographic, clinical and pharmacogenetic variables together explained 63% of the overall inter-patient variability in warfarin dose requirement in an admixed Omani patient cohort. This locally developed model performed better than the IWPC model as the latter could only explain 34% of the inter-patient variability in Omani patients^50^.

In addition to the distribution of actionable genotypes, it is also important to understand the prescription pattern of medications to prioritize the gene-drug pairs for clinical implementation. We observed that some of the CPIC Level A drugs were not prescribed by the major healthcare provider in Qatar. Thus, it may be recommended that genes, which affect the response to drugs, such as atazanavir and ivacaftor (*UGT1A1* and *CFTR*) should be given the lowest priority when implementing comprehensive pharmacogenomic testing in Qatar. This also points towards the need for concerted efforts in Qatar and other countries to develop guidelines for clinical implementation of pharmacogenes that affect drugs, which are highly prescribed in these countries and have lower efficacy and safety profiles. Such efforts will complement and contribute to the activities of CPIC, DPWG and other international consortia.

Even though the analysis of the whole-genome sequencing data from more than 6000 Qataris was intended to understand the landscape of clinically actionable pharmacogenomic variants in the population, this analysis has also generated the pharmacogenomic profile of each individual for all these drug-gene combinations. Thus, our study also provides an interesting opportunity, following further discussions with clinicians and healthcare authorities, to implement the first step toward pre-emptive pharmacogenomics in the country by returning the results to the participants or inclusion of the pharmacogenomics report of each individual in their electronic health records for use anytime during their visit to the hospitals.

A limitation of this study is the use of translation tables for genotype/diplotype generation, and their prediction of phenotypes developed based on the literature, which is dominated by studies from European or other populations, and not from the Middle East. Although it can be safely assumed that the effects of the diplotypes should be similar in different populations, the role of population-specific rare variants in the inter-individual variability of drug response is being recognized widely^52^. We have also observed novel combinations of variants in some of the genes in certain individuals, which are not currently mapped into ‘star alleles’, and hence phenotype prediction is not possible unless further functional characterization of these variants is performed.

In conclusion, we presented the first comprehensive analysis of the distribution of actionable variation in clinically important genes affecting drug efficacy or safety in the Qatari population and subpopulations. We hope that this will not only fill the gap in the literature, but also help in the implementation of precision medicine in Qatar and beyond.

## Methods

### Study samples

The study population consisted of an observational longitudinal cohort of 6218 apparently healthy adult Qatari individuals, consented and recruited by the Qatar Biobank (QBB)^46^, and whose genomes were sequenced as part of the first phase of the Qatar Genome Program (QGP). The cohort included 3528 women (56.7%), and the mean age of the participants at the start of the study was 40.2 years (SD: ±12.66). A recent publication on the genome-wide association of several traits in the same cohort provides more details of the dataset^53^. In the present study, only anonymized datasets were accessed and used for the analysis after obtaining approval from the QBB Institutional Review Board (E/2017/QGP-RES-PUB-008/0014).

### Whole-genome sequencing data

Methods used for whole-genome sequencing (WGS) and bioinformatics data processing to identify genetic variants in the cohort are described in detail elsewhere^53^ and briefly described here. As part of the QGP, the blood samples collected by the QBB were sequenced using Illumina short-read technology on HiSeq X machines to an average coverage of 30x. The raw WGS data were processed through the standard Burrows-Wheeler (BWA) algorithm (v0.7.12)^54^ and Genome Analysis Toolkit (GATK, v3.4)^55^ pipelines, following the GATK best practices for processing the data. A single multisample VCF file with all the samples sequenced and generated using the GRCh37 human genome reference was used for the analysis. After quality control, 8 samples were removed for excess heterozygosity, one for low-call rates, 65 for gender mismatch, 87 for population outliers, and 10 for identical matching^53^. Therefore, WGS data from 6045 participants were taken forward for analysis.

Copy number variation (CNV) analysis was performed using Canvas (v1.11.0)^56^ and structural variant (SV) analysis was performed using Manta (v0.29.6)^57^ and Delly (v0.7.8)^58^.

### Phenotypic data

Phenotypic data available for the QBB participants included age, sex, anthropometric measurements, self-reported questionnaire data on health conditions, lifestyle, diet, and medications.

### Selection of pharmacogenes

First, we selected all the variants known to be associated with response to drugs as annotated by PharmGKB (using clinical_ann_metadata.tsv file in clinicalAnnotations.zip downloaded from https://www.pharmgkb.org/downloads/ on 15 Dec 2020) and extracted the allele frequencies in the QGP dataset. Only simple variants (SNVs and indels) with a dbSNP rsID were analyzed in this stage and included 2629 variants in 1026 genes known to affect 559 drugs or class of drugs as annotated by PharmGKB. We also performed a detailed analysis of clinically important pharmacogenes for which annotations were made available by CPIC for their interaction with specific drugs. We analyzed 17 genes affecting 48 drugs with CPIC Level A annotation (Accessed from https://cpicpgx.org/genes-drugs/ on 15 Dec 2020).

### Pharmacogenomic analysis

Pharmacogenes with CPIC Level A annotations and one or a few variants influencing response to drugs (*CACNA1S*, *IFNL3*, *RYR1*, and *VKORC1*), we calculated the allele and genotype frequencies in the QGP population from the allele counts and allele numbers directly. For highly polymorphic genes (*CYP2B6*, *CYP2C9*, *CYP2C19, CYP2D6*, *CYP3A5*, *CYP4F2*, *DPYD*, *NUDT15*, *SLCO1B1*, *TPMT*, and *UGT1A1*), we developed custom Python scripts that took the multisample VCF as input and converted the combination of variants to haplotypes as ‘star alleles’ based on the gene-specific allele definition tables for each pharmacogene created by PharmVar, CPIC and PharmGKB. Computational phasing was performed to assign the variants to maternally or paternally derived chromosomes before the star allele assignment. The individual VCF files for each of the genes of interest were extracted using bcftools (http://samtools.github.io/bcftools/bcftools.html) from the WGS multisample VCF file. The reference-based phasing algorithm, Eagle2 (v 2.4)^59^ was used to phase the haplotypes for each of the individual genes. The genetic map file (genetic_map_hg19_withX.txt.gz) with recombination frequencies was used as a reference for the phasing algorithm.

All possible diplotypes were calculated based on the star alleles (haplotypes) detected on both the homologous chromosomes. Suballeles were considered together with the main alleles for the diplotype calculation. The CPIC diplotype-phenotype translation tables for each gene were used for diplotype to phenotype mapping and assessing their priority when implemented in electronic health records (EHR), which was used to deem the phenotype as ‘actionable’. Thus, diplotypes with the EHR priority notation ‘Abnormal/Priority/High Risk’ were considered as actionable diplotypes.

Genotype frequencies were combined when the homozygous alternate and heterozygous genotypes were predicted to lead to actionable phenotypes. Similarly, actionable diplotype frequencies were derived from the combination of multiple diplotypes predicted to lead to actionable phenotypes as described above. For example, CYP2B6 poor and intermediate metabolizers have an increased risk of adverse events when treated with efavirenz. Hence actionable frequencies were reported as combined frequencies for poor and intermediate metabolizer diplotypes for *CYP2B6*. The metabolizer phenotype can itself be derived from multiple diplotypes. For example, the diplotypes *6/*6, *6/*9, *6/*18, *6/*36, and *18/*20 present in our dataset were considered as CYP2B6 poor metabolizers based on the CPIC annotation. Details of the actionable diplotypes in each gene present in the population are provided in Supplementary Data S1–S13.

Since the CPIC star allele definition tables do not cover all the combinations of the included variants, some individuals were predicted to have ‘novel’ star alleles for certain genes, in which case, we assigned them with an ‘uncertain’ phenotype.

For the identification of the haplotypes and diplotypes of *CYP2D6* in the dataset, Cyrius v1.1 was also employed since our standard analysis was unable to call all the star alleles. Cyrius is a recent tool for specifically genotyping *CYP2D6* from short-read genome sequencing data, which is capable of alleviating the problematic alignment with *CYP2D7*, as well as utilizing information from structural variants^60^.

For the analysis of HLA genes, we used the Population Reference Graph (PRG) framework^61^. We identified HLA alleles at 6-digit allelic resolution which takes into account both nonsynonymous and synonymous single-nucleotide variants in the protein-coding region of the HLA genes.

The distribution of frequencies of the actionable variants in the overall Qatari population as well as in the Qatari subpopulations were calculated. As described elsewhere, population stratification using principal components and admixture analysis have identified six distinct subpopulation clusters within the Qatari population with different ethnicity backgrounds: Peninsular Arabs (PAR: 1,052; 17.4%), General Arabs (GAR: 2,311; 38.2%), West Eurasian/Persians (WEP: 1,372; 22.7%), Africans (AFR: 92; 1.5%), South Asians (SAS: 38; 0.6%) and Admixed (ADM: 1,180; 19.5%)^53^.

We also analyzed 2,504 genomes from the thousand genomes phase 3 consisting of 661 African, 347 American, 504 East Asian, 503 European, 489 South Asian samples^62^ through the same pipeline for all the genes except *CYP2D6* and HLA genes, for which we calculated the actionable genotypes/diplotypes from previously published studies^60,63^.

### Statistical analysis

For each variant known to be associated with drug response as annotated by PharmGKB, we applied a two-proportions z-test to identify statistically significant variants between QGP and gnomAD genomes v 3.1.1. For each gene in the study of CPIC genes, we applied a two-proportions z-test to compare the observed proportions in the two groups either QGP vs thousand genomes or within the Qatari subpopulations. In all cases, we corrected for multiple hypotheses testing using a Bonferroni procedure. All genes with an adjusted *p* value < 0.05 were considered as having different proportions in the two groups. Analyses were run using R software version 4.0.4 and graphs plotted using Prism 9 for Mac OS.

The fixation index, F_ST,_ was calculated for understanding the genetic distance between the subpopulations based on the pharmacogenes. We extracted variants found in CPIC genes from original VCF files based on location coordinates of CPIC genes. Hail (v 0.2.45) was used for the extraction of the VCF file. Then the VCF file was filtered for variant missingness of 0.01 and those failed the Hardy Weinberg equilibrium test. Plink 2.0 was used for calculating F_ST_ based on the predefined subpopulation clusters.

### Warfarin dosing

We predicted weekly warfarin dosage (mg/week) requirements for all the individuals in the cohort using the International Warfarin Pharmacogenetic Consortium (IWPC) algorithm as shown in Supplementary Data S14^42^. The race was considered as Asian for individuals in the QGP South Asian subpopulation, Black or African American for individuals in the QGP African subpopulation, and the rest were considered Missing or Mixed race. Enzyme inducer status (whether taking carbamazepine, phenytoin, rifampin, or rifampicin) and amiodarone status were obtained from questionnaire data on self-reported medications.

To compare the distribution of the predicted warfarin dosages in the Qatari population with the European population, we analysed the dosages calculated for patients recruited in the EU-PACT trial^64^. Stable dosages available for 325 patients from both arms in this trial were plotted along with the Qatari population data.

### Formulary drugs

The availability of CPIC Level A drugs for prescription in the Hamad Medical Corporation (HMC), the primary provider of health services and treatment in Qatar, was checked in the drug formulary database, which was accessed on the 7th January 2021.

### Reporting Summary

Further information on research design is available in the Nature Research Reporting Summary linked to this article.

## Supplementary information

Datasets

Reporting Summary

## Data Availability

The informed consent given by the study participants does not cover posting of participant level phenotype and genotype data of Qatar Biobank/Qatar Genome Project in public databases. However, access to QBB/QGP data can be obtained through an established ISO-certified process by submitting a project request at https://www.qatarbiobank.org.qa/research/how-to-apply-new which is subject to approval by the QBB IRB committee.
