# Supplementary material for: A population study of clinically actionable genetic variation affecting drug response from the Middle East
Source: NPJ Genom Med. 2022 Feb 15;7:10. doi: 10.1038/s41525-022-00281-5 (PMC8847489; doi:10.1038/s41525-022-00281-5)
Supplement: Supplementary file 2 — Reporting Summary [file 41525_2022_281_MOESM2_ESM.pdf]

nature portfolio

Corresponding author(s): Puthen Veettil Jithesh

Last updated by author(s): Oct 14, 2021

Reporting Summary

Nature Portfolio wishes to improve the reproducibility of the work that we publish. This form provides structure for consistency and transparency in reporting. For further information on Nature Portfolio policies, see our Editorial Policies and the Editorial Policy Checklist.

Statistics

For all statistical analyses, confirm that the following items are present in the figure legend, table legend, main text, or Methods section.

n/a	Confirmed	
☐	☒	The exact sample size (n) for each experimental group/condition, given as a discrete number and unit of measurement
☐	☒	A statement on whether measurements were taken from distinct samples or whether the same sample was measured repeatedly
☐	☒	The statistical test(s) used AND whether they are one- or two-sided<br>*Only common tests should be described solely by name; describe more complex techniques in the Methods section.*
☐	☒	A description of all covariates tested
☐	☒	A description of any assumptions or corrections, such as tests of normality and adjustment for multiple comparisons
☐	☒	A full description of the statistical parameters including central tendency (e.g. means) or other basic estimates (e.g. regression coefficient) AND variation (e.g. standard deviation) or associated estimates of uncertainty (e.g. confidence intervals)
☐	☒	For null hypothesis testing, the test statistic (e.g. F, t, r) with confidence intervals, effect sizes, degrees of freedom and P value noted<br>*Give P values as exact values whenever suitable.*
☒	☐	For Bayesian analysis, information on the choice of priors and Markov chain Monte Carlo settings
☒	☐	For hierarchical and complex designs, identification of the appropriate level for tests and full reporting of outcomes
☒	☐	Estimates of effect sizes (e.g. Cohen's d, Pearson's r), indicating how they were calculated

Our web collection on statistics for biologists contains articles on many of the points above.

Software and code

Policy information about availability of computer code

Data collection	Illumina HiSeq X Software (v3.0)
Data analysis	BWA-mem (v0.7.12), GATK (v3.4), Canvas (v1.11.0), Manta (v0.29.6), Delly (v0.7.8), Eagle2 (v 2.4), Cyrius (v1.1), R (v4.0.4), Prism (v9 for Mac OS), Plink (v2.0), Hail (v 0.2.45)

For manuscripts utilizing custom algorithms or software that are central to the research but not yet described in published literature, software must be made available to editors and reviewers. We strongly encourage code deposition in a community repository (e.g. GitHub). See the Nature Portfolio guidelines for submitting code & software for further information.

Data

Policy information about availability of data

All manuscripts must include a data availability statement. This statement should provide the following information, where applicable:

- Accession codes, unique identifiers, or web links for publicly available datasets
- A description of any restrictions on data availability
- For clinical datasets or third party data, please ensure that the statement adheres to our policy

Access to the data used in this study will be controlled access through a dedicated portal by QGP as per its policy. The informed consent given by the study participants does not cover posting of participant level phenotype and genotype data of Qatar Biobank/Qatar Genome Project in public databases. However, access to QBB/QGP data can be obtained through an established ISO-certified process by submitting a project request at https://www.qatarbiobank.org.qa/research/how-to-apply-new which is subject to approval by the QBB IRB committee

March 2021

Field-specific reporting

Please select the one below that is the best fit for your research. If you are not sure, read the appropriate sections before making your selection.

☒ Life sciences ☐ Behavioural & social sciences ☐ Ecological, evolutionary & environmental sciences

For a reference copy of the document with all sections, see nature.com/documents/nr-reporting-summary-flat.pdf

Life sciences study design

All studies must disclose on these points even when the disclosure is negative.

Sample size	This is Qatar Genome project Phase 1 study of whole genome sequence of 6,218 Qatar Biobank (QBB) participants, recruited by the QBB. We used all the data made available to us as end users and we had no control to do any sample size calculation.
Data exclusions	We applied standard per-determined quality control measures which included the excluded of samples with excess heterozygosity (n=8), duplicates (n=10), call rate <95% (n=1), gender ambiguity (n=65), and population outliers (n=87). Two individuals not belonging to any of the major subpopulations were also removed. After exclusions, the final clean set (n=6,045) was used for analysis. The median sequencing depth was 32X.
Replication	We used all the samples from the cohort for this study without exclusions for calculation of the frequencies.
Randomization	The randomization is not relevant in this study. This is a population-based cohort study.
Blinding	The blinding is not relevant in this study. This is a longitudinal population-based cohort study.

Reporting for specific materials, systems and methods

We require information from authors about some types of materials, experimental systems and methods used in many studies. Here, indicate whether each material, system or method listed is relevant to your study. If you are not sure if a list item applies to your research, read the appropriate section before selecting a response.

Materials & experimental systems

n/a	Involved in the study	
☒	☐	Antibodies
☒	☐	Eukaryotic cell lines
☒	☐	Palaeontology and archaeology
☒	☐	Animals and other organisms
☐	☒	Human research participants
☒	☐	Clinical data
☒	☐	Dual use research of concern

Methods

n/a	Involved in the study	
☒	☐	ChIP-seq
☒	☐	Flow cytometry
☒	☐	MRI-based neuroimaging

Human research participants

Policy information about studies involving human research participants

Population characteristics	The present study was performed on the Qatar Biobank (QBB) study participants. Individuals are eligible to participate in the study if they are Qatari nationals aged 18 years and older. The age range was 18 to 88 years with an average of 40.2 years (SD=12.7). 56.7 % of the participants were females whereas 43.3% were males. Detailed description of the QBB participants was published previously
Recruitment	This is a population-based cohort study. Apparently healthy Qatari national 18 years or older are recruited by the Qatar Biobank.
Ethics oversight	All Qatar Biobank (QBB) participants signed an Informed Consent Form prior to their participation; QBB study protocol ethical approval was obtained from the Hamad Medical Corporation Ethics Committee in 2011 and continued with QBB Institutional Review Board (IRB) from 2017 onwards and it is renewed on an annual basis

Note that full information on the approval of the study protocol must also be provided in the manuscript.
